# Next preventive strategies for oral health: evolution or revolution?

**DOI:** 10.3389/fpubh.2023.1265319

**Published:** 2023-10-05

**Authors:** Denis Bourgeois

**Affiliations:** Health Systemic Process Research Unit UR 4129, University Claude Bernard Lyon 1, University of Lyon, Lyon, France

**Keywords:** policy, prophylaxis, microbiome, gingivitis, fluoride, interdental brush, hygiene, biofilm

The globalization of sugar consumption since the 1950s has resulted in dental caries becoming one of the most common chronic diseases worldwide. Sugars have pervaded society and should be considered the driving force behind caries and obesity (1). In response to this epidemiological challenge, in 1978, the World Health Organization (WHO) resolution on fluoridation and dental health (WHA31.50) highlighted that “where fluctuation of public drinking water supplies is not feasible for technical or other reasons, other methods of ensuring optimal daily fluoride application—fluoride toothpaste (FT)—or intake should be considered” (2). Currently, FT is part of the WHO model list of essential medicines and is also used therapeutically for the inactivation of incipient carious lesions (3). Twice-daily brushing was recommended. A direct consequence of the fluoride WHO initiative, notably to deliver fluoride in saliva, was the exponential growth of the toothbrush market.

This revolution, which conflicted with the interests of dental professionals in the Western curative model of care, was presented as the most cost-effective, evidence-based, and realistic strategy with an ambitious objective: to reduce the incidence of caries among 12-year-olds in industrialized countries by 90% by the year 2000 and to neutralize the adverse effects of sugar on oral health. In the 1990s, caries, or at least the process of caries, was under control for most people in industrialized countries. However, evolution is still necessary to fight caries, especially in children. FT may not be a realistic option in low-income countries (4).

Although oral health is a fundamental human right and is inseparable and indivisible from overall health and wellbeing, the treatment of oral diseases and conditions is often cost-prohibitive and not part of universal health coverage (5). In the existing model of oral disease prevention, an action such as toothbrushing with FT is frequently a solitary activity undertaken by an individual away from supporting relationships and networks (6). In addition, disease treatment was and still is the primary aim of dentists. Dentists have also taken little interest in advocacy to promote good oral health, preferring to treat rather than prevent oral diseases (7). The cost to society of managing the consequences and complications of tooth decay, mainly in the adult and senior populations, is still estimated to be approximately US$ 387 billion annually in direct costs (8). Thus, the development of clearer and more transparent conflicts of interest policies and procedures to limit and clarify the influence of the sugar industry on research, policy, and practice is needed. Public health policies for oral and other non-communicable diseases should prioritize addressing the commercial interests (9) of the sweetened food and beverage industry, dental product manufacturers, and dental research organizations, in that order (10).

Deficiencies in oral health prevention, health promotion, and care are particularly pronounced among adolescents and young adults. The burden of specific oral diseases, especially plaque-induced gingivitis, is very high for this generation (11, 12). In fact, the current poor situation in terms of plaque-induced gingivitis and interproximal caries results primarily from inadequate oral hygiene. The transition from a cariogenic to a periodontal microbiota at the end of adolescence requires the definition of individual prevention policies (13). Although still necessary, the fluoride policy is no longer the first priority for this generation. The quality of brushing on the accessible surface with respect to brushing expectations is the top priority. The goals are to mechanically disorganize the biofilm, mainly located in the gingival sulcus and at the mucogingival junction (14). In 2023, the current challenge is how to achieve lifelong oral health for young adults who have benefited from fluoride policies in childhood, who adopt oral hygiene and cleanliness behaviors, and who, in the majority of cases, visit the dentist for check-ups.

Currently, one of the problems is the lack of consensus on recommendations for toothbrushing techniques and cleaning devices among oral health professionals, including, unfortunately, dental companies. Excessive variability in many aspects of the design and methodology of selected studies hinders conclusions on an ideal manual or power toothbrushing technique (15). How to optimize the mechanical disorganization of the biofilm within the scope of individual prophylaxis is a challenge that requires evolution and the mobilization of clinical research.

Toothbrushes cannot access the interdental spaces, which represent 40% of dental surfaces. In clinically healthy young adults, a type I embrasure is defined as a closed interdental space filled with interdental papilla and is the most commonly seen. The presence of major periodontal pathogens (*Porphyromonas gingivalis*) in type I spaces has been demonstrated and quantified (16). Low-grade chronic inflammation from the earliest age may be a reason, with lifelong exposure that contributes to periodontal diseases and to many human diseases that were previously not considered inflammatory disorders, including diabetes, cardiovascular diseases, rheumatoid arthritis, cancer, and chronic obstructive pulmonary disease (17).

Regarding the use of a toothbrush on accessible surfaces, the daily use of interdental brushes (IDBs) is essential for interspace biofilm removal, substantially reducing gingival bleeding inflammation, the leading symptom of plaque-induced gingivitis, and achieving a safe and high standard of interdental cleaning (14). A lack of daily home proximal cleaning makes the implementation of adequate oral hygiene difficult. IDBs should be recommended as an effective alternative to traditional dental floss and considered the first choice for interproximal cleaning. A small-diameter (0.6 to 0.7 mm) IDB should be recommended as the first choice for interproximal cleaning as long as the size of the interdental embrasure space allows its passive insertion (16). However, for larger embrasures, it would be necessary to choose an IDB with a larger calibrated diameter.

The question is how to develop new strategies to disseminate in a short period when it took 30 years to modify hygiene behaviors using fluoride toothpastes that have reached their ethical and health limits. Following the example of the initial WHO resolution, a similar position should be taken with regard to the daily use of interdental brushes in healthy adolescents and young adults. Strategies designed to manage inflammation need to incorporate interproximal cleaning tools/methods on a routine basis (16). Interdental hygiene requirements are very high, even among healthy people. Making people and professionals realize that adequate toothbrushing without interdental brushing is unrealistic requires a veritable revolution to change mentalities and behaviors.

How can we meet expectations? What guidance could motivate students, dentists, patients, and the population to take better care of their oral health? At the end of the process, screening of the accessibility of interdental spaces should be a component of routine examinations for all patients, contributing to an integrated approach to chronic disease prevention to reduce exposure to major risk factors (16). However, continuing professional development does not seem to be the best choice for successfully implementing this strategy. The dental profession is not ready to change its practice model, which will involve moving to more complex funding mechanisms for oral health as opposed to the traditional restorative care model (6). This raises questions about how this will work in practice and how it can be measured. Changes in the scope of practice will give rise to further questions and concerns. Who will deliver what aspect of oral health promotion and prevention, who will oversee it, who will be paid, how will they be paid, and by whom? Finally, the implications of more evenly distributed funding will need to be considered (6).

It is time to take radical action and implement innovative individual oral prophylaxis strategies for healthy young adults. Evidence-based guidelines and simple and cost-effective preventive approaches exist, but they need to be rigorously promoted and implemented (18). Of course, communicating these current concepts as well as the role of the oral microbiome in oral and general health among researchers, clinicians, and policymakers is part of the strategy (1, 19). However, academic education may have the most direct effect on the process, which must include an innovative investment in the curricula of dental faculties from the earliest stages of study. Dental students must become key players in the education of individual dental prophylaxis. To do so, they need to integrate interdental hygiene into their daily oral hygiene routine so that they are aware of the obstacles and levers. This will enable them to pass on their knowledge, skills, and practices to their future patients in an appropriate manner. Furthermore, they must free themselves from the pressure of brands, which have no place in healthcare and education.

The next contribution, which is grounded in the United Nations' Transforming our World: the 2030 Agenda for Sustainable Development and its 17 Sustainable Development Goals (SDGs), in particular, SDG Goal 3 (ensure healthy lives and promote wellbeing for all at all ages), will be to achieve wellbeing for all through public health approaches and interprofessional and transdisciplinary education involving future health workers, including physicians, nurses, pediatricians, and pharmacists; this will be the effective foundation of an intersectoral collaboration to achieve greater oral health equity (6, 9).

## Author contributions

DB: Conceptualization, Investigation, Methodology, Writing—original draft, Writing—review and editing.
